# Recent Developments in Single-Cell Metabolomics by Mass Spectrometry—A Perspective

**DOI:** 10.1021/acs.jproteome.4c00646

**Published:** 2024-10-22

**Authors:** Boryana Petrova, Arzu Tugce Guler

**Affiliations:** † Medical University of Vienna, Vienna 1090, Austria; ‡ Department of Pathology, Boston Children’s Hospital, Boston, Massachusetts 02115, United States; § Institute for Experiential AI, Northeastern University, Boston, Massachusetts 02115, United States

**Keywords:** metabolomics, mass spectrometry, single-cell, multiomics, metabolic imaging, cellular heterogeneity

## Abstract

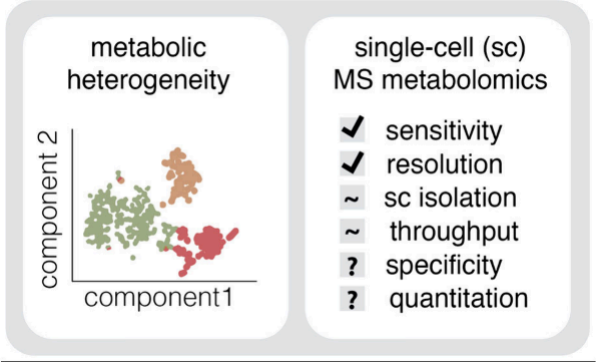

Recent advancements in single-cell (sc) resolution analyses, particularly in sc transcriptomics and sc proteomics, have revolutionized our ability to probe and understand cellular heterogeneity. The study of metabolism through small molecules, metabolomics, provides an additional level of information otherwise unattainable by transcriptomics or proteomics by shedding light on the metabolic pathways that translate gene expression into functional outcomes. Metabolic heterogeneity, critical in health and disease, impacts developmental outcomes, disease progression, and treatment responses. However, dedicated approaches probing the sc metabolome have not reached the maturity of other sc omics technologies. Over the past decade, innovations in sc metabolomics have addressed some of the practical limitations, including cell isolation, signal sensitivity, and throughput. To fully exploit their potential in biological research, however, remaining challenges must be thoroughly addressed. Additionally, integrating sc metabolomics with orthogonal sc techniques will be required to validate relevant results and gain systems-level understanding. This perspective offers a broad-stroke overview of recent mass spectrometry (MS)-based sc metabolomics advancements, focusing on ongoing challenges from a biologist’s viewpoint, aimed at addressing pertinent and innovative biological questions. Additionally, we emphasize the use of orthogonal approaches and showcase biological systems that these sophisticated methodologies are apt to explore.

## 1 Introduction

Cell-to-cell variability, also known as cellular heterogeneity, is an intrinsic feature of cell populations, organs, and tumors and is driven by transcriptional,^1^ metabolic,^2,3^ epigenetic,^4^ and proteomic^5^ profiles. High-throughput sc resolution analyses like sc transcriptomics^6,7^ or sc proteomics^1000−9,112^ have successfully captured multiple aspects of cellular heterogeneity and significantly advanced our understanding of cancer biology^10,11^ immunology,^12^ and the study of normal and pathological states of tissues.^13−16^

Metabolomics is the study of small molecules (metabolites), which are components in cellular metabolism, serving as substrates, products, and intermediates in biochemical reactions.^17,18^ Beyond being integral to all cellular function, metabolism governs physiological^19,20^ and disease states,^21,19,22,23^ including cell differentiation,^243^ proliferation,^3^ and response to therapy.^24^ Changes in metabolites can accurately and quantitatively reflect changes in cellular states where metabolic heterogeneity is encountered frequently.^3,25,26^ Thus, metabolomics is crucial for capturing the dynamic phenotype of individual cells.

Multiomics integrative analyses can provide a more powerful and comprehensive profile of a cell’s state^27−32^ and enhance our understanding of underlying cellular dynamics. Functional cellular heterogeneity can be observed even within genetically identical cell populations.^33,34^ Neither protein abundance nor metabolome abundance can be directly inferred from mRNA levels.^35,36^ For example, sc proteomic studies have identified developmental fates and cellular states that are not captured by sc RNA-seq^37^ and have also revealed many proteins that are not predicted by the genetic code.^38^ Orthogonal approaches can complement^27−32^ and validate each other and access mechanistic details beyond linear regulatory programs. Additionally, confounding factors like cell cycle stages, cell types, or activation states may be better elucidated through one data modality that then complements and reinforces the others.

To integrate omics and build comprehensive models of cellular heterogeneity, quantitative high-throughput data^39,40^ is needed. Currently, metabolism is not commonly captured at a quantitative sc level. Cell-to-cell variability also necessitates significant depth of analysis to confidently capture rare cellular phenotypes. The sc transcriptomics and sc proteomics already being high-throughput methods argues for the development of dedicated methods to quantitatively access cellular metabolic heterogeneity at equivalent throughputs.

High-resolution mass spectrometry (HRMS), using mass analyzers like Fourier transform ion cyclotron resonance (FTICR), orbitrap, and time-of-flight (TOF) alongside live-cell sampling techniques^41−52^ and imaging-based approaches^53−55^ with matrix-assisted laser desorption/ionization mass spectrometry (MALDI-MS) have advanced the field of metabolomics. These technologies facilitate de novo discovery, quantitative and high-throughput measurements, and have significantly improved the signal-to-noise ratio required for sc analysis. However, these analytical methods also pose several obstacles for comprehensive sc analysis^53−60^ and several hurdles remain that are currently rarely addressed in sc metabolomics approaches.

This perspective examines latest advancements in sc metabolomics by MS from the viewpoint of a biologist. Novel methodologies, particularly in ionization and high-throughput sc isolation strategies, as well as enhancements in MS imaging (MSI) are scrutinized for their ability to answer pertinent biological questions. Key remaining challenges in capturing biological heterogeneity are emphasized which future technology developments can address. Additionally, orthogonal techniques that provide complementary insights into underlying mechanisms and corroborate biological findings are highlighted. Finally, biological areas that could significantly benefit from sc technologies are reviewed.

## 2 Metabolic Heterogeneity

**Figure 1:**
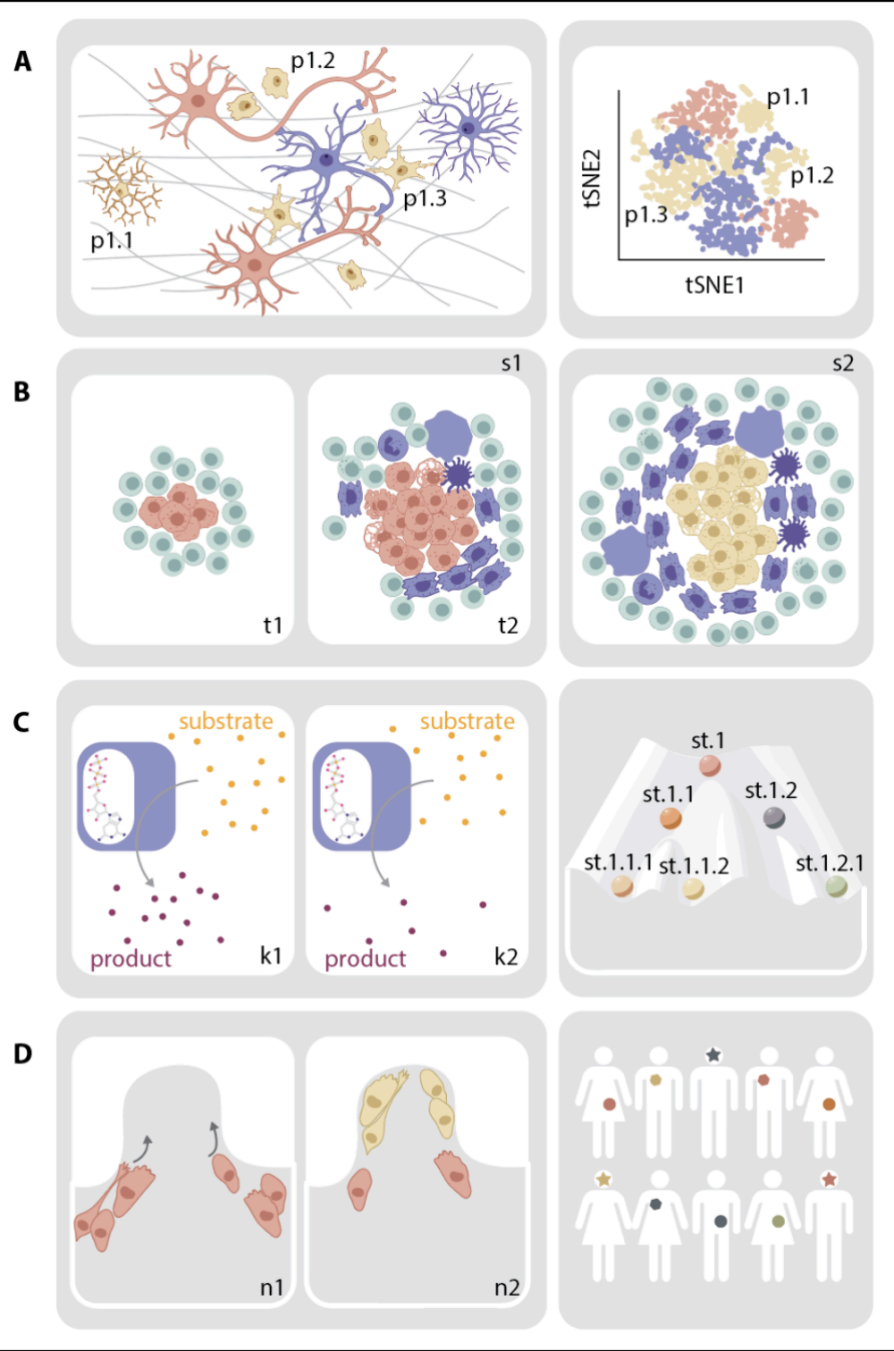
Schematic representation of the types of observed metabolic heterogeneities in biological systems. (A) The figure illustrates the complexity of tissue architecture in the brain, highlighting interactions between distinct cell types and their subpopulations (population 1 with subtype 1–3: p1.1; p1.2; p1.3). These interactions contribute to the formation of an intricate, heterogeneous tissue structure as can be captured by sc technologies. (B) Heterogeneity can arise from spatial variations within tissues such as depicted for region s1 or s2. Temporal fluctuations are also depicted, such as at time point t1 or t2 during tumor growth and immune cell infiltration. Tumor cells are in red or yellow, diverse immune cells are in blue and green (C) Stochastic variations in enzyme expression and catalysis contribute significantly to metabolic diversity. Here different rates of catalytic reactions are depicted as k1 and k2 (left panel). Further, a Waddington’s Landscape of metabolic states is represented (right panel) with different quasi-stable metabolic states designated as “st”. (D) Depicted are intercellular differences (left panel), between cells within different microenvironmental niches, each designated as n1 or n2. The right panel depicts interorganismal and interindividual variability.

Metabolic heterogeneity is a multifaceted phenomenon^61^ reflecting the diversity of metabolic states within and across cells, organisms, and populations, influenced by various factors across space and time (Figure 1). This heterogeneity can arise within the same tissue, such as the brain, where interactions between distinct cell types and their subpopulations contribute to a complex tissue architecture (Figure 1A). Further heterogeneity arises from spatial variations in tissues, such as tumors where different regions may exhibit distinct metabolic profiles due to variations in oxygen and nutrient availability (Figure 1B). Temporally, metabolic states can fluctuate due to changes in environmental conditions or during different stages of cell growth or organismal development, aging, and immune responses (Figure 1B). Metabolic enzymes are often regulated at the level of substrate availability or product inhibition rather than transcriptionally,^62^ and lowly expressed enzymes can significantly impact metabolite concentrations through high fluxes. In addition, stochastic variations in enzyme expression and catalysis contribute significantly to metabolic diversity even within genetically identical populations^63^ (Figure 1C). Such variations could further be seen analogous to the Waddington’s Landscape—metaphorically representing a ball rolling down a contoured landscape with multiple valleys and peaks which depict how gene regulation guides cells through various developmental pathways toward distinct fates. Similarly, metabolic heterogeneities in space and time could determine stable steady states in different cells and thus guide cells toward distinct metabolic fates (Figure 1C).

Heterogeneity is further compounded by intercellular differences, where cells within the same tissue may adapt differently based on microenvironmental niches,^64,65^ and interorganismal and interindividual variability that encompasses broader biological and environmental variations (Figure 1D). In sum, metabolic heterogeneity is not just a reflection of a preexisting transcriptional or proteomic diversity and metabolite concentrations are influenced by both intrinsic genetic controls and extrinsic environmental factors. Thus, metabolic heterogeneity reflects real-time biochemical activities and adaptations, offering an independent readout of the functional state of cells.

For further discussions on metabolic heterogeneity, we refer the reader to recent reviews on aspects of tumor^3,66^ or developmental heterogeneity.^66,67^ While microorganismal systems further exhibit clinically relevant metabolic heterogeneities, these will be beyond the scope of the current perspective, and we refer the reader to a recent review by Evans and Zhang.^68^

### 2.1 Strategies and Considerations for Effective Capture of Metabolic Heterogeneity

**Figure 2:**
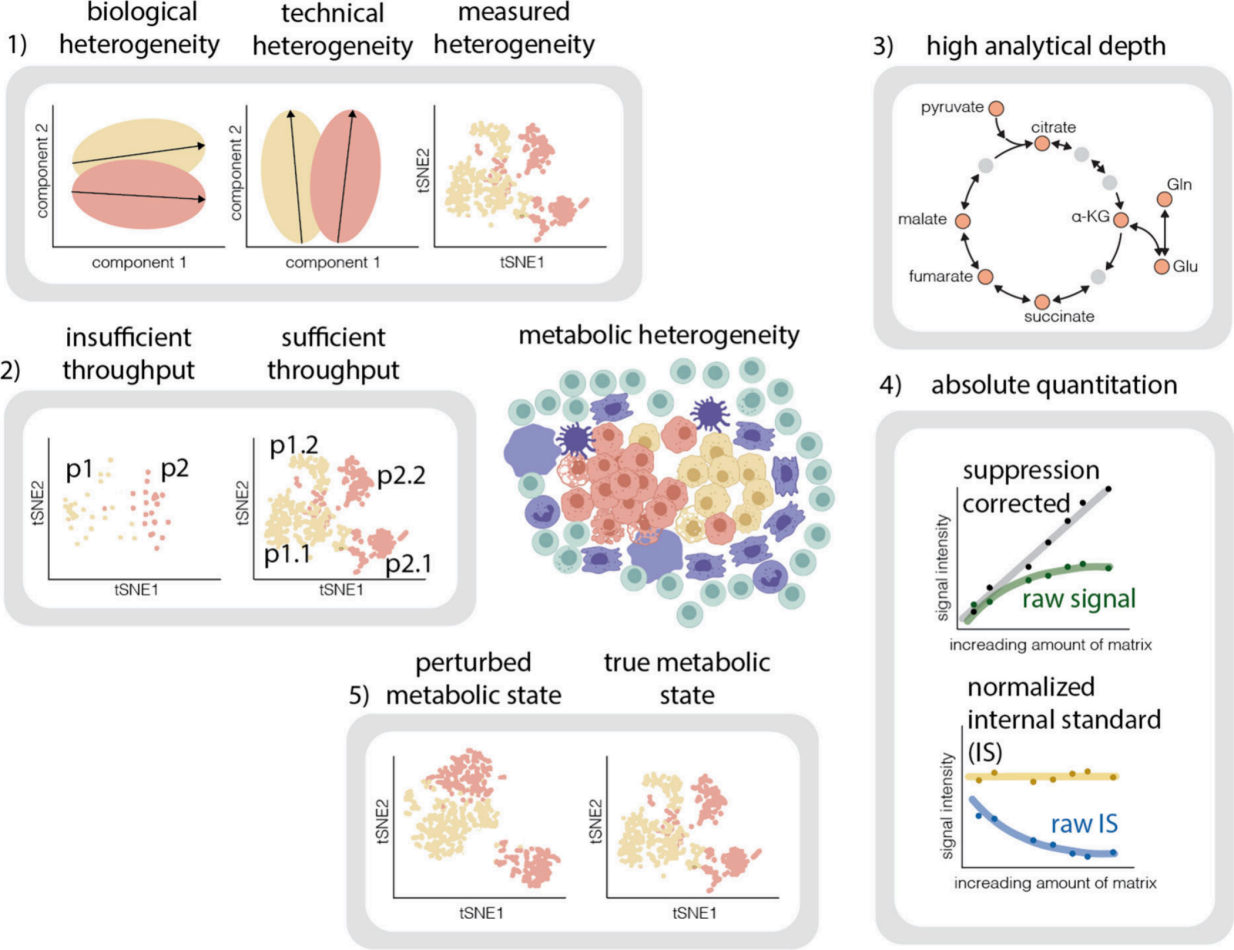
Schematic representation of different considerations for effective capturing of metabolic heterogeneity. Highlighted are 1) confounding between biological heterogeneity and technical noise; 2) necessity to achieve sufficient throughput; 3) analytical depth; 4) quantitation and 5) reliability in preserving true metabolic states. Dimensionality reduction graphs are represented with Component 1/2 and tSNE1/2 axis. Abbreviations include p: population; Gln: glutamine; Glu: glutamate; IS: internal standard.

Capturing and interpreting heterogeneity involves several important considerations (Figure 2). One shared among sc omics is making sure that measured heterogeneity is revealing true biological heterogeneity. Bulk experiments average out technical noise along with biological heterogeneity, while sc methods are sensitive to and thus confounded by both.^69−71^ On the flip side, sc metabolomics technologies must achieve sufficient analytical depth, throughput, and quantitation to reliably capture cellular metabolic heterogeneity. Another important consideration is in ensuring that captured states reflect true metabolic states. Due to its highly dynamic nature, metabolism requires more rigorous preservation methods than those used in sc transcriptomics or sc proteomics. These considerations interplay with the specific technical capabilities of MS that lead to unique challenges with sc metabolomics.

To make sure sc metabolomics can capture true biological heterogeneity, methods need to be able to distinguish cellular metabolites from serendipitous contaminants and noise, particularly at the low concentrations typical in sc analysis or for transiently produced metabolites under unique conditions. In metabolomics, the absence of a comprehensive blueprint of cellular metabolites leads to the exploration of a wide chemical space, unlike genomic or protein databases, which have well-defined analyte search spaces. This search space is further amplified by a plethora of adducts and fragments which could even form in a sample-dependent manner.^72^

This challenge is especially relevant to MS metabolomics techniques that do not use analyte separation prior to detection, such as direct infusion mass spectrometry (DIMS), flow injection mass spectrometry (FI-MS), or MSI. Unlike LC-MS and GC-MS, which use chromatography to separate metabolites, providing high selectivity and greater certainty in compound annotations,^73^ DIMS and MSI bypass this step.^74^ Further, techniques like MALDI-MS often necessitate harsh sample preparation that can alter the native metabolite profiles^75^ and the matrices used in sample preparation can introduce additional chemical contaminants. Significantly, it has been shown that even in bulk MS metabolomics, the majority of detected signals are from nonbiological origin.^76^ Thus, despite added certainty MS^2^ analysis and separation techniques alone cannot confirm the biological origin of annotated compounds. Therefore, metabolite annotations must be validated with orthogonal methods or confirmed with rigorous mock samples and parallel isotope labeling, as done in metabolite credentialing.^76,77^

In addition to ensuring specificity, sc metabolomics methods must achieve sufficient depth of analysis to inform on metabolic heterogeneity. “Depth” refers to the number of metabolites consistently detected across single cells, but the optimal informative depth remains unclear. In bulk metabolomics, quantifying approximately 100–150 central carbon metabolites is often sufficient to provide insights into hypothesis-driven biological questions. Interestingly, sc RNA sequencing studies have also shown that analyzing a fraction of expressed transcripts—around 400 genes, can be sufficient to capture meaningful biological insights.^78^ Therefore, prioritizing quantitative accuracy for a specific set of metabolites over broadening the depth could be more beneficial and should be considered within the context of clear biological questions.

Beyond depth of analysis, scalability is a significant consideration in sc metabolomics. The majority of current technologies struggle to process large numbers of cells efficiently. As an example, methods involving chromatography^79,80^ or live-cell sampling rarely achieve high-throughput analysis.^45−52^ Multiple recent techniques demonstrate significant advances^81−87^ and are able to access cellular heterogeneity across many cells as well as biological replicates. Nonetheless, the optimal number of cells should be tested independently, guided by statistical considerations based on the heterogeneity within the biological context and the hypothesis being investigated.^88^

For sc metabolomics to achieve reliable quantitation, satisfactory solutions to the “matrix effect” must be found.^89,90^ The matrix effect refers to the difference in mass spectrometric response for an analyte in standard solution versus the response for the same analyte in a biological matrix.^91^ This effect compromises the linearity of MS signals due to either ion suppression, where ionization efficiency is reduced bycompetiting of charges during ionization, or ion enhancement, where one analyte increases the ionization of an another. These effects vary for individual compounds and cannot be uniformly corrected, ultimately obscuring biologically significant variations.

The matrixes used in MALDI-MS—compounds that cocrystallize with the analyte molecules and assists the ionization by absorbing laser energy, vaporizing, and helping transfer protons to the analyte molecules—arguably lead to stronger matrix effects.^53,92^ Importantly, however, ion suppression is a broad challenge that ultimately obscures quantitation in all ionization methods.

Ion suppression effects can cause the metabolic state of samples, cell types, or tissue regions to confound results, highlighting the need for independent control measures. To address this challenge, the implementation of normalization strategies using isotopically labeled external standards is considered the method of choice.^93−95^ Recently some attempts to predict ion suppression have been proposed^96,97^ and are currently being commercialized.^98^ Overall, ion suppression correction is imperative when quantitative assessment of any complex matrix is desired.

Another primary challenge in sc metabolomics is the highly dynamic nature of metabolism. On the one hand, there are biology-intrinsic considerations: metabolite concentrations can fluctuate dramatically within narrow time frames both due to internal stochastic cellular processes or external environmental fluctuations. On the other hand, there are technology specific considerations—certain cell isolation techniques can cause changes in the metabolome due to stress or introduction of reactive chemicals.^99,100^ For example, metabolic fluxes could be perturbed within seconds^101,102^ and glucose labeling kinetics in mammalian systems have shown that the entire glucose-derived pool of NADH could be turned over within 15 min.^103^ Therefore, capturing unperturbed metabolomes requires careful consideration of these intrinsic and extrinsic factors and future advancements (as discussed in section 7) should offer rigorous validation strategies.

Overall, the distinct challenges of sc metabolomics necessitate ongoing advancements in analytical technologies and methodologies to accurately capture the complex metabolic landscapes of individual cells. Effective solutions have addressed some challenges, such as sensitivity and scalability. Advances in sc proteomics can inspire further improvements.^104,105^ For example, data collection strategies like data independent aqcuisition parallel accumulation–serial fragmentation (DIA-PASEF), prioritized Single-Cell ProtEomics (pSCoPE), and sequential window acquisition of all theoretical mass spectra (SWATH) analysis have increased the depth of sc proteome analysis.^106−109^ Progress is also being made in isolating unperturbed metabolomes, with further advances potentially drawing inspiration from sc proteomics, where ready-to-use solutions have enhanced method transferability and reproducibility, making it easier to establish sc proteomics in new laboratories.^110−112^ However, challenges like specificity and quantitation remain largely unresolved, requiring dedicated sc metabolomics approaches. We would ultimately need technologies that comprehensively address all these challenges. If a technology solves the scalability challenge but does not address the specificity challenge, biological interpretation will be limited. Striking a balance between achieving high specificity and sensitivity, high accuracy and quantitative outputs, preserving the native state of cellular metabolites during analysis, and attaining high-throughput, high-resolution data remains a critical goal that will shape the future trajectory of this research field.

### 2.2 Applications of ML for Effectively Capturing Metabolic Heterogeneity

**Figure 3:**
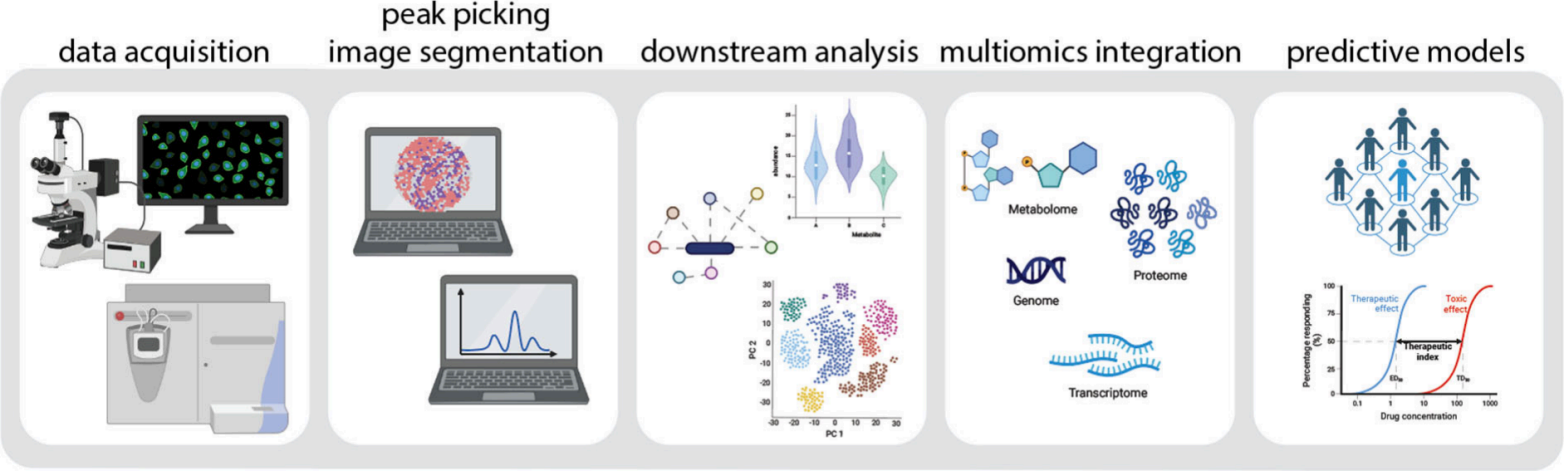
Schematic representation of the various stages where ML methods could be applied in sc metabolomics: data acquisition, peak picking and image segmentation, downstream analysis, multiomics integration, and predictive models. ML methods could be used in upstream data analysis, such as peak picking, *in situ* image segmentation for subcellular metabolomics, and intelligent cell isolation. Nonlinear relationships in diverse metabolomics data sets could be solved by applying ML. ML also assists in integrating multiomics data and building predictive models for drug responses, especially in cancer research. Unsupervised and supervised learning methods are essential for clustering and classifying single cells, profiling biomarkers, assessing chemotherapy resistance, and identifying clinical risk phenotypes.

Machine learning (ML) techniques will increasingly be used to enhance capturing cellular heterogeneity and to support method development for reproducible data extraction and analysis pipelines (Figure 3). One example is the application of ML to resolve nonlinear relationships in diverse metabolomics data sets.^113^ Further, mixture models and multivariate analysis are robust computational methods often applied to the highly variable sc data sets.^114,115^ Unsupervised and supervised learning methods are commonly applied for clustering and classifying single cells. Some applications of these methods include clustering of single cells into meaningful populations when labeled data is unavailable,^116^ classification of cellular compositions in patient-derived tissues for identifying sc phenotypes and assessing clinical risk,^117^ profiling multiple secreted biomarkers for tumor cell classification^118^ and monitoring chemotherapy resistance.^119^

ML algorithms also play a crucial role in analyzing upstream raw experimental data or image analysis. MSI has become a powerful tool for sc proteomics and metabolomics by allowing spatially resolved analysis at the sc level and requires specialized data analysis that frequently use ML methods.^120^ ML methods are applied in areas ranging from *in situ* image segmentation for subcellular metabolomics,^121^ intelligent cell isolation^122^ to broader workflows in MSI data analysis.^123^ These ML applications have simplified and standardized various aspects of sc metabolomics.

Future research will surely see an expansion of ML applications. Automated feature extraction with ML will support handling vast amounts of future sc high-throughput data. Further use of ML for sc would be in building models for predicting drug response to treatments, especially in cancer research.^124^ ML methods could also assist in multiomics integration of sc metabolomics information to map omics data onto a metabolomics model and aggregate different omics layers with multilayer network methods.^125^ Ongoing improvements in the application of ML methods will undoubtedly continue to benefit sc metabolomics.

## 3 Methodology Advancements

Analysis of single cells is not a new concept, having first emerged in the 1970s. Early technological developments in the 1970s included the introduction of laser ablation mass spectrometry for biological sample analysis.^126^ Jimenez et al. used MALDI-MS for direct peptide fingerprinting of single neurons in Lymnaea brain, demonstrating its potential application to sc studies.^127^ Some of the first sc metabolomics studies were conducted by Kennedy and Jorgenson in the late 1980s, who analyzed the amino acid composition of a single giant neuron from the snail *Helix aspersa* using open tubular capillary chromatography.^128^ Around the same time, Wallingford and Ewing analyzed neuronal cell contents from *Aplysia californica* using capillary sampling.^129^ Further significant momentum was gained in the 1990s due to advancements in molecular biology and imaging techniques.^130,131^ In the 1990s, Masujima’s development of the “video-mass spectroscope” allowed for live sc analysis using a glass capillary and nanoelectrospray ionization.^132^ Although these methods were initially low-throughput, further research has led to more robust and versatile techniques.^133^ Today, advanced technologies enable high-throughput analysis of thousands of cells, with techniques offering the sensitivity to analyze even the smallest single cells, including individual bacteria.^87,134,135^

HRMS such as those based on Orbitrap and TOF instruments, have become indispensable when it comes to capturing metabolic sc heterogeneity. Despite significant advancements, challenges in capturing sc metabolic heterogeneity persist due to specific technological limitations of the instrumentation and the inherent complexities of the biological systems and analytes.

Below we present some of the latest innovations that are pushing the boundaries of how we capture and analyze the complex metabolic landscapes of individual cells.

### 3.1 Advancements in Ionization Strategies for Enhanced sc Metabolomics

Detecting low-abundance metabolites in minute volumes has driven the development of highly sensitive MS detectors. Sensitivity issues in sc metabolomics persist due to chemical class-specific challenges that need dedicated solutions. For example, techniques like MALDI and nanoelectrospray ionization (nanoESI) face challenges with low ionization efficiency for nonpolar compounds and analyte class-specific effects that can skew metabolic profiling.^136,137^ Further, MALDI’s reliance on matrix selection can lead to matrix effects that alter the ionization efficiency and specificity for individual compounds.^138,139^ This has motivated the development of novel, more sensitive and comprehensive ionization technologies.

Recent improvements in laser-based ionization methods have driven significant progress in sc metabolomics, enhancing sensitivity and specificity. The “LAESI microscope,″ utilizing laser-ablation electrospray ionization mass spectrometry (LAESI-MS), enables spatially resolved MS analysis of single cells.^140−143^ This method has successfully identified chemical species specific to physical structures in *Fittonia argyroneura* leaves with high spatial resolution, demonstrating its capability to pinpoint metabolic variations within isolated single cells or tissues.^141^

Further extending the capabilities of LAESI, recent developments have incorporated ion mobility (f-LAESI-IMS-MS),^142^ substantially enhancing metabolite detection. This innovation produced 259 sample-related peaks per cell, nearly doubling the 131 sample-related peaks per cell achieved by traditional f-LAESI-MS. By fully automating the *in situ* sampling platform, the technique reached an overall sampling rate of 804 cells per hour. Applied to the study of soybean (*Glycine max*) root nodules, this method revealed both unimodal and bimodal metabolite abundance distributions, underscoring its potential to elucidate complex metabolic heterogeneities within single cells and across cell populations.^142^ Although powerful, the LAESI-MS approach lacks chromatographic separation, which can lead to ion suppression effects and inability to distinguish isomeric and isobaric compounds.^144^ Future developments should aim to address these issues to enable reliable quantitative analysis.

Beyond these laser-based innovations, the development of nanosecond pulsed dielectric barrier discharge ionization^145,146^ (DBDI) reported another significant signal improvement. DBDI utilizes high-voltage pulses as narrow as 100 ns with a delay of about 900 μs to significantly reduce background chemical noise and enhance ion signal. This technique has achieved improvements in signal-to-noise ratios and sensitivity by up to 172% and 271%, respectively, enabling more sensitive detection of volatile molecules in complex mixtures.^145^

For ionization techniques specifically tailored to sc analysis, a hybrid ionization source combining nanoESI and DBDI offers an innovative approach for detecting metabolites with varying polarities.^146^ A capillary with a 1 μm inner diameter tip samples cells and serves as the nanoESI source to ionize polar metabolites, while the DBDI source improves the ionization of apolar metabolites. The hybrid mode detected a broader range of metabolites in onion and human pancreatic cancer cell line PANC-1 cells, enhancing coverage, ionization efficiency, and detection limits. Another innovation on an ionization technique this time tailored to a specific chemical class of analytes was described: the online quaternized derivatization platform. By desorbing metabolites with a laser and reacting them online with a derivatization reagent transmitted by carbon fiber ionization (LACFI-MSI), this platform notably increased the mass signals of monoglycerides and diglycerides, thereby improving the sensitivity and specificity of glyceride profiling.^147^ These compound-specific effects should be carefully considered when selecting these techniques to address specific biological questions.

To broaden the scope of the chemical space probed by a single MS technique, a novel concentric hybrid ionization source, combining nanoESI and atmospheric pressure chemical ionization (nanoESI-APCI), has been designed to simultaneously detect polar and nonpolar metabolites in single cells. This source has improved the limit of detection by an order of magnitude to 10 pg mL–1. After optimizing operational parameters, 254 metabolites detected in nanoESI-APCI were tentatively identified, demonstrating its application in studying the metabolic heterogeneity of the human hepatocellular carcinoma tissue microenvironment. This was united with laser capture microdissection (LCM), facilitating a throughput of 80 cells per minute and discrimination of cancer cell types and subtypes, and exploring metabolic perturbations due to glucose starvation in Michigan Cancer Foundation-7 (MCF7) cells as well as the metabolic regulation of cancer stem cells.^83^ A separate study focused on refining the analysis of lipid-based metabolites. Researchers developed an on-probe derivatization technique and combined it with noncontact nanocarbon fiber ionization tailored for the sensitive detection of fatty alcohols and sterol metabolites at the sc level.^148^ This method utilized a unique ionization source that is compatible with low-polarity solvents such as dichloromethane, enhancing detection capabilities for these challenging analytes. Additionally, proton-transfer-reaction (PTR) mass spectrometry, traditionally used for detecting trace levels of volatile organic compounds, is now being adapted for the analysis of small nonvolatile molecules. Employing supercritical fluid extraction (SFE), this approach allowed for rapid and selective extraction of lipophilic compounds from complex matrices. The combination of PTR MS with SFE has shown potential for analyzing small molecules in single cells, particularly lipophilic compounds, with the method observing significant numbers of ions in both positive and negative ion modes.^149,150^ The ability to match several of these ions to chemical formulas from the LipidMaps database underscored the method’s potential for detailed lipid analysis at the cellular level. It will be interesting to determine whether the observed improvements in ionization are specific to certain lipid classes and if they are compatible with broader quantitative analysis of lipids in complex tissues and matrices.

As an alternative to the widely used LC technique for metabolite separation, capillary electrophoresis (CE) provides unique advantages for sc metabolomics.^43,151−153^ It requires very small sample volumes (nanoliter to picoliter range), making it ideal for analyzing limited or precious samples and typically has shorter analysis times, allowing for quicker separation and analysis. Utilizing nano capillary electrophoresis–mass spectrometry (nanoCEMS), researchers have optimized sheathless ionization and sample flow rates to further optimize CE-MS technology for the sc level of analysis. This allowed for efficient ionization through an ESI mechanism using a tapered tip, quantifying 20 amino acids from 10 individual cells.^154^ Researchers further introduced a sample enrichment method, large-volume dual preconcentration by isotachophoresis and stacking (LDIS) and applied to the nanoCESI-MS. Compared with normal sheathless CE-MS, coupling of nanoCESI and LDIS provided up to 800-fold increase of sensitivity. Analogously, the field amplified sample injection (FASI) CE-ESI-MS method provided a 100- to 300-fold improvement in detection limits over hydrodynamic injections.^153^ In FASI, the sample is injected into the capillary under conditions that cause a field-induced concentration at the beginning of the capillary. This method further employed internal standards which enhanced analyte identification and quantification accuracy. However, the limited number of cells that can be accessed through these technologies, particularly due to difficulties to automate sample loading, restricts their biological applicability, requiring innovative sampling approaches to increase throughput.

Several groups have reported novel ionization strategies coupled to sc manipulation or isolation. In this regard, the 3D-printed ionization source, integrated sample introduction, metabolite extraction, and ionization into a single device for sc MS analysis. This simplified sc analysis and improved measurement reproducibility, with the probe adaptable for different cell sizes. In a follow up study, it was used for high-throughput analysis of three types of cancer cells to distinguish them based on detected metabolites.^155^ A further follow-up study employed ML algorithms to identify significant differences in metabolic features among 756 single cells.^156^ The development of intact living-cell electrolaunching ionization mass spectrometry^82,124^ (ILCEI-MS) similarly addressed sc isolation and sensitivity improvement into one technological advancement. It used a capillary emitter to transport entire living cells directly into the MS ion-transfer tube, achieving a high detection throughput without solvent use, thus minimizing dilution and matrix interference.^82^ Importantly, the technique allowed for relatively high-throughput of 51 cells per min and reported analysis of more than 4000 primary single cells digested from fresh multiorgan tissues of mice, demonstrating its applicability and reliability. The rapid isolation of single living cells likely preserves their metabolomes intact; however, future studies should formally validate this assumption using markers for oxidation, stress, or cellular energy status.

The methodologies outlined here present important advancements in sensitivity and ionization efficiency. These advancements showcase the diverse innovations in ionization strategies, from improving signal detection to tailoring methods for sc analysis. However, limitations like throughput, ion suppression, and preservation of metabolic states persist, highlighting areas for future development and integration with sc isolation pipelines. In addition, although these technologies showcase successful capture of technical heterogeneities, there is a need for further validation of biological results. It remains to be seen if these novel techniques are prone to quantitative biases due to sample intrinsic and metabolic-state-specific ionization effects for example. It is also unclear whether biological heterogeneities, beyond those distinguishing different cell types, can be effectively captured. Nevertheless, the achieved sensitivity and throughput have significantly advanced the sc MS technology.

### 3.2 Advances in sc Mass Spectrometry Imaging Techniques

Recent advancements in sc MSI technologies have greatly improved our understanding of metabolic heterogeneity at the cellular level.^157^

#### 3.2.1 Improvements to Sensitivity and Resolution for MSI for sc Metabolomics

MALDI stands out for its gentle ionization and is still the most widespread MSI scanning method.^158,159^ Nevertheless MALDI-based ionization could present limitations such as invasive sample preparation or limited suitability for smaller molecules. Recently, a plethora or techniques are emerging as alternatives or improvements. For example, transmission ambient pressure laser desorption ionization (t-AP-LDI) with post photoionization (PI) offers high spatial resolution for analyzing lipids and neurotransmitters, promising submicron resolution in the future.^160^ The laser-based ionization technique termed fiber-based laser ablation electrospray ionization (f-LAESI) further facilitate high-resolution profiling of single cells.^142,161,162^ For example, application of this method, utilizing mid-infrared ablation for sampling, uncovered metabolic subpopulations and enabled the analysis of infection dynamics in plants.^161^ The ability to sample single cells directly, without prior isolation, preserved the metabolome more effectively. Additionally, the use of ultrahigh resolution made it possible to determine elemental formulas for unknown metabolites, particularly those produced by rare cells. This approach has revealed hidden subpopulations within a multicellular symbiotic organ, showcasing bimodal chemical distributions that indicate cells in proliferating and quiescent phases—a distinction only possible through sc analysis. Future work on ambient analysis methods, such as LAESI or t-AP-LDI MS, could focus on limiting the effects of high background interference to ensure quantitative analysis. Additionally, the spectral complexity inherent to MSI techniques due to the lack of orthogonal separation, could be addressed by incorporating ion mobility-based separation and high-resolution MS.^163,164^

Several recent developments, though not yet at a sc level, show promise and motivate future investigations. Advances in MALDI Fourier transform ion cyclotron resonance mass spectrometry imaging (FT-ICR MSI) have revealed metabolic heterogeneity in adrenocortical carcinoma and human epidermal growth factor receptor 2 (HER2)-positive gastric cancer.^165,166^ This innovation enables detection of metabolic variability at both inter- and intratumor levels and linked it to treatment outcomes and the efficacy of chemotherapy. Although specific metabolite changes were not validated in terms of biological origin of MS signal or its quantitative accuracy, the heterogeneity observed at the level of tumor subpopulations was demonstrated as an independent prognostic factor, underscoring the potential clinical utility of such analysis. Another novel imaging technique demonstrated tissue heterogeneity on the example of a mouse hippocampus. Researchers developed a novel laser microdissection-coupled shotgun lipidomic platform,^167^ which pushed the boundaries of quantitative and broad-range lipidome analysis toward sc spatial resolution. This involved preparation of successive cryosections from tissue samples, cross-referencing of native and stained images, laser microdissection of regions of interest, in situ lipid extraction, and quantitative shotgun lipidomics. Researchers demonstrated a quantitative limit at about 10 cells. It remains to be seen if further improvements will allow these techniques to reach quantitative levels at resolutions required for sc analysis.

To enhance the sensitivity and resolution of spatial metabolomics a novel approach using segmented temperature-controlled desorption electrospray ionization (STC-DESI) mass spectrometry was developed which precisely controlled desorption and ionization temperatures.^168^ This method concentrated the spray plume and accelerated solvent evaporation at varying temperatures, achieving a spatial resolution of 20 μm. The achieved resolution allowed for the direct observation of heterogeneity around individual amyloid-beta (Aβ) plaques in brain tissue, revealing crucial details like carnosine depletion in a mouse model of Alzheimer’s disease. Although this approach targeted micron-scale features, and not single cells per se, approximately 150 μm in size, the significant boost in sensitivity allowed detection of low-abundance metabolites and less ionizable neutral lipids, bridging the gap toward sc resolutions.

Several further techniques showed excellent image resolution and suitability for sc metabolomics. The Ambient Fiber-Assisted Desorption Electrospray Ionization Mass Spectrometry Imaging (AFADESI-MSI) technique allowed for direct, in situ analysis of biological tissues under ambient conditions without the need for sample preparation.^169−172^ This should present an advantage for sc metabolomics by potentially increasing the chance of sampling unperturbed metabolomes. Application of AFADESI-MSI allowed spatial metabolomics studies of specific brain regions in diabetic encephalopathy model rats.^169^ A related technique, secondary ion mass spectrometry (SIMS), was combined with TOF (TOF-SIMS) and provided high-resolution insights into glioblastoma, detecting proteins and lipids from the same sample with an 800 nm resolution.^173^ Of note, while both SIMS and AFADESI-MSI are used for surface analysis and imaging, SIMS requires vacuum conditions and can cause more sample damage, whereas AFADESI-MSI operates under ambient conditions and is considered generally less invasive although more prone to environmental interferences.

Nanostructure imaging mass spectrometry (NIMS) and NIMS with fluorinated gold nanoparticles (f-AuNPs), have been proposed to overcome the challenges of high ionization noise and low metabolome coverage of traditional MALDI, offering comprehensive, ultrasensitive, and high-resolution MSI of metabolites in biological systems.^174,175^ In an example application tailored to sc metabolomics, NIMS with f-AuNPs permitted the simultaneous detection of polar metabolites and lipids in a single and cohesive analytical session, and subsequently allowed the systems-level interpretation of metabolic changes.^174^ Despite these advantages, AuNPs require high laser energies for desorption/ionization, which can cause laser-induced fragmentation and metabolite adsorption on the nanoparticle surface, leading to bias in the annotation and quantification of biologically relevant small molecules.^176^

#### 3.2.2 Strategies for sc Targeting with Multimodal Mass Spectrometry (MMS) for High-Throughput Lipidomics and Metabolomics

MSI-based techniques can be effectively applied to dissociated and isolated single cells, however, traditionally, a significant amount of time is spent sampling areas between dispersed cells or analyzing only part of a cell, depending on its position within the imaging raster pattern. Recent advancements have integrated MSI with segmentation strategies, enabling higher throughput and improved targeting of specific cell types or rare cell populations in tissue and culture models.^177^

A high-throughput MMS approach analyzed 154,910 sc lipidomic profiles from various brain regions, identifying lipid clusters and metabolic diversity across cell types and throughout development.^87^ To achieve this, researchers used a Python-guided platform and microscopy imaging to select regions of interest, performing MALDI-MS analysis only at those locations.^178^ Multiple further studies enabled by this approached have been published, extending applications into diverse imaging and ionization modalities.^177^ In a more recent approach, matrix sublimation enabled *in situ* spotting of single cells under a microscope. Further improvements combined trapped ion mobility separation with dual-polarity ionization MSI and implemented a custom developed images coregistration parser, allowing automated profiling of human PANC-1 and activated PSC cells, with 52 single cells analyzed per cell type and metabolic differences validated based on perturbations.^179^ Future work could show if this platform can be expanded to higher throughputs and more diverse cell types.

In another study, instead of using MALDI ablation marks for coregistration, cellular information was derived from selected MSI channels (i.e., spectra). High-resolution MALDI-2-MS imaging, combined with optical microscopy, allowed coregistration of mass spectrometric and optical data for hundreds of cocultured cells.^180^ This enabled statistical analysis of cell heterogeneity based on individual sc mass spectra, with 99 lipid species tentatively assigned, highlighting lipid heterogeneity across cell types and developmental stages. Although coregistration with immunofluorescence markers was demonstrated, future work could extend this method by incorporating biological validation such as specific perturbations or correlations with other sc omics or orthogonal techniques.

Further applications of microscopy guided MS for cultured cells have demonstrated the potential for analyzing rare cell populations and supporting personalized precision medicine. Combining sc MALDI-MS with immunocytochemical classification enabled high-throughput analysis of over 1,800 rodent cerebellar cells, revealing lipid heterogeneity between astrocytes and neurons, and identifying potential cell subtypes.^181^ Another example applied MALDI-MSI to Fluorescence-activated Cell Sorting (FACS)-sorted multiple myeloma (MM) and normal plasma cells, analyzing 16 cells from each group and revealing a significant decrease in PC (16:0/20:4) in MM cells.^182^ This method stabilized lipid profiles via fixation and improved sc resolution combining MSI and microscopy and enhancing the laser ablation area. However, future work should validate its advantages over bulk analysis.

Future advancements in multimodal sc MS of dissociated cells could address several key limitations, such as the loss of tissue context and potential metabolic perturbations introduced during cell isolation, as well as artifacts resulting from cell culturing. There is also a need to reduce reliance on cell fixation, which currently limits applicability to metabolomics. Incorporating controlled metabolic perturbations and refining quantitative capabilities will be crucial for uncovering subtle but biologically significant differences. Moving forward, it is crucial to balance these advancements with the method’s inherent strengths, such as reduced interference from neighboring cells, leveraging coculturing for controlled experiments and integrating orthogonal approaches like microscopy for cell identification—tailoring the methodology to address specific biological questions and hypotheses effectively. Notably, the strategies discussed above have been employed to enhance throughput and resolution in sc tissue MMS and are expected to become increasingly important in the future.^177,183−185^

#### 3.2.3 Improvements to Data Analysis and Segmentation for sc Metabolomics with MSI and MMS

Advances in MSI and MMS techniques would not have been as successful without the corresponding advances in image analysis, processing, and the development of innovative methods for cell isolation and arraying. These enhancements facilitate precise targeting, identification, and segmentation of single cells, crucial for transitioning from traditional tissue culture applications to more challenging *in situ* analyses. For instance, a study using a tapered probe for pneumatically assisted nanoDESI optimized the conditions for handling arrayed single cells, achieving a throughput of about 3 cells per minute.^186^ This method also included the optimization of washing and analysis conditions, with validation efforts focusing on glucose starvation in INS-1 cells involving 93 or 97 cells, showcasing its effectiveness in processing isolated single cells under specific experimental conditions. Similarly, another study introduced a rapid and sensitive technique using infrared matrix-assisted laser desorption electrospray ionization mass spectrometry^187^ (IR-MALDESI-MS) to investigate lipid profiles of isolated Henrietta Lacks (HeLa) cells. This approach managed to identify 45 distinct lipid species across 34 analyzed cells. The integration of RastirX, a MATLAB-based control software, and a microscope-linked camera is anticipated to further reduce the data acquisition time to less than 1 s per cell, thus enhancing throughput and the overall efficiency of sc lipidomic analyses.^187^ Moreover, a high-throughput approach utilizing a microarray-based preparation workflow was demonstrated in a study where lipid and pigment composition were analyzed in single algae cells. This method successfully analyzed over a thousand individual cells, highlighting the substantial heterogeneity among them and underscoring the capacity of MSI to handle large-scale sc studies effectively.^81^

Further addressing the challenges of segmentation and integration in tissue imaging, a multimodal MSI strategy has been developed to MALDI-MSI with confocal immunofluorescence imaging.^188^ This integrative pipeline coregisters MALDI-MSI data with confocal images labeled with pluripotency antibodies and Hoechst nuclei dye, facilitating precise alignment and segmentation. Such alignment permits the extraction of MALDI spectral abundances on an approximate cell-by-cell basis, enabling the analysis of cell-specific metabolic signatures. Despite some signal spillover beyond segmented areas, suggesting challenges in achieving exact sc resolution, this approach provides a comprehensive data set suitable for multivariate analysis techniques that uncover metabolic spatial relationships within the data.

A related technology, SpaceM, integrated in situ MALDI ablation signals with fluorescence-based cell segmentation, allowing for signal coregistration and rapid automated sc analysis of individual or mixed cell populations.^134^ Although this approach offered high throughput, cosampling of neighboring cells potentially obscured subtle differences and reduced quantitative accuracy. Later improvements addressed these limitations,^184,189^ and future work could further rely on isotopically labeled internal standards to resolve potential differences in ionization efficiencies between cell types, enhancing quantitative accuracy.

Another innovative MMS segmentation approach employed deep learning to reshape the design-build-test-learn (DBTL) cycle: RespectM,^84^ based on discontinuous mass spectrometry imaging, could detect more than 700 metabolites (primarily lipids) at a rate of 500 cells per hour (acquiring 4,321 sc level metabolomics data points). RespectM distinguished single microbial cells from the blank matrix with an accuracy of 98.4%.^190^ The reported method could also classify *Chlamydomonas reinhardtii* single cells among allelic strains. By further employing laser etching guided droplet microarray (LEM), 2,5-dihydroxybenzoic acid (DHB) matrix sublimation, and sparse data matrix generation, researchers improved on signal cell discrimination and peak generation challenges relative to traditional MALDI-based approaches. To address cross-contamination between adjacent rasters, which reduces the confidence of MSI data—a phenomenon aggravated by the small size of microbial cells—researchers used the discontinuous MSI acquisition function of Bruker’s flex imaging. It will be important to follow up on this study and address if matrix application affected individual cells metabolomes and quantitative accuracy.

Advances in MSI and MMS techniques continue to refine our ability to observe and analyze metabolic heterogeneity at or near-sc resolutions within complex tissue environments. Despite high resolution below sc size, these methods still need to contend with the complex 3D tissue architectures and further advances in intelligent segmentation will be needed. For example, although SpaceM, a method for in situ sc metabolomics, is powerful and adept at mapping pixela-by-pixel correlations of metabolic targets within the cytosolic boundaries of large cells in cultures, it encounters resolution challenges when applied to tissue sections.^134^ The spatial single nuclear metabolomics SEAM method used nuclear regions as segmentation guide to probe the nuclear metabolomic profile within the native tissue environment. While SEAM enabled submicron metabolite mapping in cells and tissues, it predominantly extracted nuclear patterns, leaving the association of specific cell types with metabolic profiles less defined and omitting broader cytosolic boundaries.^191^

#### 3.2.4 3D Imaging and Multiomics Integration with MSI

Advances in MSI sc metabolomics techniques have enabled integration with other omics technologies. Incorporating these details can link enzymatic activities and cellular identities to metabolic variations, deepening the understanding of biochemical processes and their impact on health and disease.

A recent study integrating sc MALDI-MSI and Visium 10x RNAseq exemplifies the strength of these correlative measurements, though not strictly at the sc level. While primarily focused on cancer-specific alterations across human sample cross sections, the study successfully demonstrated the potential of MSI to correlate product, substrate, and enzyme changes, highlighting the utility of integrating various omics approaches.^192^ This represents a step forward in using MSI for complex biological analysis.

Further advancements have been achieved by combining MALDI-MSI with immunofluorescence microscopy, which enhanced in-tissue spatial resolution. This approach precisely mapped metabolic changes within tissues and employed a Spatial Coherence Measure (SCM) to distinguish real spatial patterns from noise in ion distributions, thus enhancing the robustness of spatial metabolomics. An antibody labeling strategy identified different cell types, providing a histological structure based on complementary insights from metabolite distributions.^193^ However, the exclusion of matrix effect normalization in this analysis indicates that future validations are necessary to confirm individual metabolic alterations.

The Single Cell Spatially resolved Metabolic (scSpaMet) framework represents a significant innovation by combining untargeted spatial metabolomics with targeted multiplexed protein imaging to profile single immune and cancer cells in human tissues. Developed through an extensive platform for image analysis and omics integration, this approach uses deep learning-based joint embedding to reveal unique metabolite states within cell types and local metabolite competition among neighboring single cells. Incorporating the 3D spatially resolved metabolomic profiling (3D-SMF) for submicron resolution metabolic imaging with multiplex IMC proteomic imaging, scSpaMet correlates over 200 metabolic markers with 25 protein markers in individual cells within native tissues, demonstrating its effectiveness across various crowded human tissues.^183^ Despite limitations due to harsh sample preparation and the invasive nature of metabolite analysis, this framework could become a powerful tool for understanding cell-type-specific metabolic profiles and their implications in tissue heterogeneity and disease.

Expanding beyond these single tissue sections or isolated cultured cells, 3D-SMF initially introduced by Ganesh, S. et al. enhanced cell specificity by using an isotope-tagged antibody library to label tonsil tissues, which were then spatially correlated and classified in three dimensions.^194^ Likewise, infrared laser ablation atmospheric pressure photoionization mass spectrometry^195^ (LAAPPIMS) has shown promising potential for depth profiling analysis. This technique achieved 70 μm lateral resolution, enabling detailed analysis of *Arabidopsis thaliana* leaf substructures at various levels of the leaf tissue. LAAPPIMS effectively mapped analytes at different depths, distinctly resolving the topmost trichomes and cuticular wax layer from the underlying tissues and revealing varied distributions of metabolites across different leaf parts such as veins, cuticle, and sc trichomes.^196^ However, imitations remain as LAAPPIMS signals could vary due to ion suppression and the nature of the sample surface, which can lead to loss in quantitative accuracy and reduced reproducibility—-critical for correct biological interpretation. Nevertheless, these advancements collectively broaden the scope of MSI from mere surface analysis to a comprehensive three-dimensional exploration of metabolic functions within and across cell types.

Overall innovations in sc MSI highlight the breadth of technological advances, each tailored to unique applications. However, while these innovations have enhanced the capabilities of sc metabolomics, they often face challenges such as no adequate controls for the preservation of metabolic states or for presence of contaminants arising from sample preparation or interference from neighboring cells or the extracellular matrix. Furthermore, matrix effects that affect quantification and annotation necessitate further validation. We need strategies to corroborate MSI findings applying orthogonal techniques (as discussed in section 6), normalization controls, and further insights from multiomics integration. For example, matrix effects in techniques such as MALDI could be validated for individual metabolites using ^13^C-labeled standards infused with the matrix.^92^ We currently do not have clear understanding of the limitation of newer technologies. This highlights the need for improved standardized strategies for quantitative accuracy.

### 3.3 Innovative Techniques for Direct sc Sampling and Extraction

The sc isolation and extraction are critical for metabolomics research, as detailed in recent reviews.^100,197^ Taking inspiration from live video microscopy, patch clamping or capillary sampling, which can probe cellular functions noninvasively with high precision and over extended periods, several techniques were developed that sampled cytoplasm or cellular components and directly injected into a mass spectrometer.^41,44,46,49−52,54,55,57,59,198−201^ Cellular components could be extracted *in situ* into a nanoelectrospray tip or using a probe or capillary. These techniques, while pioneering, were originally limited by low throughput.

Efforts to enhance scalability have led to more automated and high-throughput methods. For instance, the computer-assisted microscopy isolation (CAMI) method combined imaging, machine learning, and high-throughput microscopy to guide cell extraction through laser microdissection or micromanipulation.^122^ The improvement in throughput, analyzing hundreds of cells, was promising but will likely need an order of magnitude increase in the future to compare with other omics applications. Similarly, a miniaturized picolitre extraction system offered automated sample preparation for sc MS analysis and could serve as inspiration for future improvements beyond the current 20 cells per hour.^202^

Methods utilizing electromigration and microaspiration have also been developed to better preserve metabolic integrity. One study used calibrated microaspiration from *Xenopus laevis* embryos to comprehensively detect metabolites,^203^ while a more recent study demonstrated utilization of this strategy for single yeast cells and applied electromigration and electroporation to release cell contents into a sealed volume for nanoESI analysis.^204^ This study’s approach offered advantages in throughput and sensitivity when single cells can be easily obtained in suspension. However, additional considerations will be required to translate this to mammalian systems, which may experience metabolic alterations during the procedure.

In conclusion, while these advancements in sc isolation and extraction techniques offer promising avenues for metabolomics research, they underscore a persistent need to address throughput limitations, and the preservation of metabolome integrity. It should be noted that direct sampling techniques offer a significant advantage over sc isolation by minimizing the introduction of external contaminants and providing better control over noncell-intrinsic signals. Nonetheless, these factors should still be carefully monitored.

### 3.4 Innovative Techniques for sc Capture by Microfluidics or Droplet Control

Microfluidics has become an essential tool for advancing sc metabolomics, providing unmatched precision in isolating and analyzing individual cells. Recent innovations such as controllable cell printing^205^ and microfluidic chips with microwell arrays^206,207^ have significantly enhanced the high-throughput monitoring and rapid chemical lysis of single cells, respectively. Reviews have also emphasized the increasing role of microfluidics in sc metabolomics, highlighting its potential to revolutionize this field.^208−210^

To enable high-precision sc isolation for metabolite profiling, the sc printer technology combined with liquid vortex capture-MS (SCP-LVC-MS) was developed. This technique dispenses sc droplets for accurate chemical analysis.^211^ The SCP-LVC-MS approach was validated by analyzing the lipid composition of *Chlamydomonas reinhardtii*, *Euglena gracilis*, and HeLa cells in their native growth media. The method successfully identified various lipids in single cells and ensured no signal carryover between cells. It differentiated mixed microalgae cells based on lipid levels. Promisingly, the results were validated by comparing quantitative peak areas to bulk lipid extracts and showed clear differences in cellular populations under nitrogen-limited and normal growth conditions. Additionally, the quantitative analysis was refined using an internal standard and expanding this strategy to include more chemicals^212^ or a broader range of standards and biological conditions will robustly validate its applicability to sc analysis.

Integrated microfluidic chips could offer a comprehensive approach by enabling multiplexed and automated processing for multiomics applications. An example is a system developed for comprehensive proteomic sample preparation combined with^213^ DIA. Although this system could face challenges with throughput and sample loss and requires further validation for sc metabolomics. Further droplet-based microfluidic systems have shown success in precisely encapsulating single cells and reducing contamination.^214^ However, their effectiveness in eliminating nonbiological contaminants has yet to be fully validated. Another droplet-based technique used a focused sheath fluid to align cells toward a droplet junction, where they were encapsulated and characterized by fluorescence signals. This method has demonstrated a potential for future implementation when combined of an electro-coalescence-based selective isolation module.^215^

High-precision microfluidic systems have enabled the isolation of single plant cells with exceptional accuracy. For instance, a microfluidic cell-picking robot has been used to isolate protoplasts from *Catharanthus roseus*, allowing for both targeted and untargeted metabolomic analysis via ultrahigh performance liquid chromatography–mass spectrometry (UPLC-MS), analyzing 586 cells.^216^ This approach not only achieved promising throughput but also showed absolute quantification of metabolites. A follow up stidy demonstrated variability among different plant tissues although it did not account for matrix effects and ion suppression.^217^

Microfluidic integration with imaging technologies has also enabled the analysis of spatiotemporal tumor heterogeneity. By merging ultrasound elastography with mass spectrometry imaging (UEg-MSI), researchers have been able to map metabolic changes in tumors both *in vivo* and *in vitro*, providing a detailed profile of tumorigenesis stages.^218^ Another study utilized a multicolor fluorescence detection-based microfluidic device (MFD-MD) to analyze primary liver cells from mice stimulated with ethanol. This system effectively separated cells and measured metabolites like hydrogen peroxide, glutathione, and cysteine, revealing significant cellular heterogeneity. These redox-sensitive molecules are crucial for assessing the metabolic state of single cells, serving as potential benchmarks for future more extensive MS-based metabolomics measurements.

These microfluidic advancements highlight the potential for precise sc manipulation and analysis, providing invaluable insights into cellular metabolic processes. Integrating these methods with further advances in MS-ionization and detection remains a challenge. Additionally, the impact of various device materials on detected metabolites and their interference with quantitative outputs needs further exploration to enhance efficiency of these technologies in sc metabolomics. Nonetheless, microfluidic advancements have the potential to provide a highly effective and advantageous cell isolation system that is quick, gentle and integratable with other omics technologies. Cells could be kept in suitable growth conditions throughout isolation up until a brief wash and extraction steps thus optimally preserving original metabolic states.

## 4 Applications of sc Metabolomics in Tumor Biology

**Figure 4:**
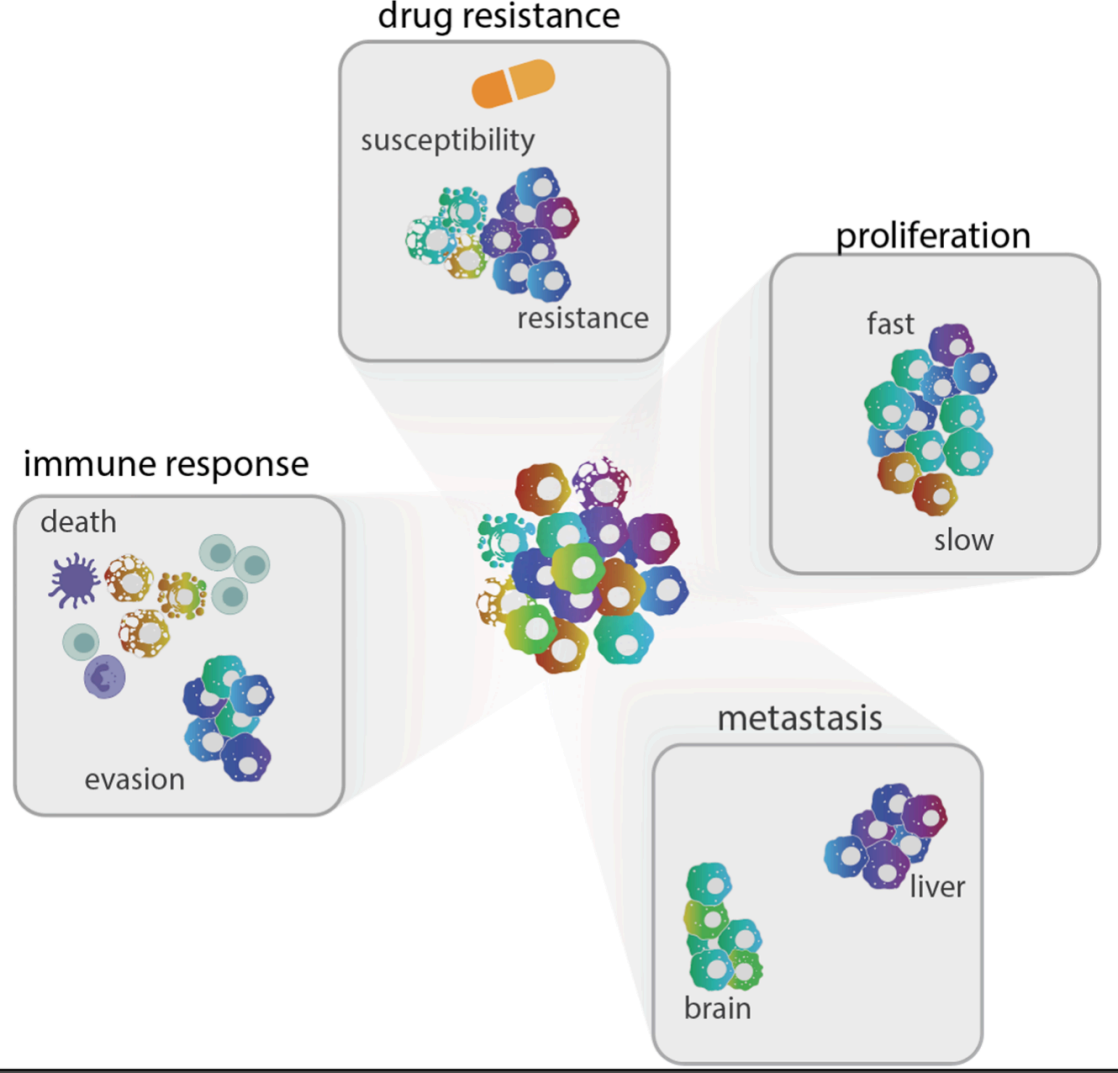
Schematic representation of metabolic heterogeneity within tumors. This diagram highlights key aspects of tumor heterogeneity influenced by metabolic states at the sc level: 1. Drug resistance, illustrating how variations in metabolic state translate to varying response to therapy; 2. Proliferation rates, illustrating how metabolic states dictate proliferation rates; 3. Metastatic potential, illustrating how tumor cells in organs like the brain or liver exhibit distinct metabolic profiles; 4. Immune response, illustrating pathways of immune evasion and cell death that arise from differences in metabolic states within cancer cells. Each cluster represents distinct cellular populations (depicted as different colors) within a tumor, emphasizing the complexity of interactions and behaviors influenced by metabolic states at the sc level.

Only through the application of sc technologies is it possible to capture and characterize rare cells such as cancer stem cells, which can be critical for metastatic potential and drug resistance. Advances in sc metabolomics have provided valuable insights into metabolic heterogeneity within tumors, enhancing our understanding of tumorigenesis, tumor progression, and treatment resistance (Figure 4). Readers interested in a more comprehensive discussion of tumor drug resistance, metabolomics, and tumor heterogeneity can refer to recent review articles.^219−221^

### 4.1 Direct Measurements of Metabolic Heterogeneity in Tumors

The sc MSI has evolved into an important tool in oncology, revealing the metabolic diversity within tumors and identifying significant metabolic differences across tumor types and regions. For example, techniques such as high-resolution MALDI-MSI have stratified gastric cancer patients and distinguished nonsmall-cell lung cancer (NSCLC) subtypes by identifying specific lipid and metabolic profiles. They highlighted metabolic alterations between cancerous tissues and surrounding stroma, demonstrating the potential of MSI to uncover both temporal and spatial tumor heterogeneity.^220,222−224^ Spatial MSI analyses in glioblastoma have for instance revealed variations in antioxidant levels and energy demands between tumor cores and peritumoral areas.^224^ Similarly, imaging mass cytometry has provided insights into tumor and immune cell architecture in diffuse large B-cell lymphoma, correlating cellular structures with chemotherapy responses,^225^ while DESI-MSI has successfully differentiated breast cancer tissue types, linking tumor grade and hormone receptor status to distinct metabolic profiles.^226^

A novel methodology expanded the capabilities of MSI for multiomics approach to tumor biology. The study utilized water gas cluster ion beam secondary ion mass spectrometry^227^ ((H_2_O)n-GCIB-SIMS) for comprehensive lipidomic and metabolomic profiling on frozen hydrated tissue sections.^228^ This approach enabled subsequent profiling of cell-type-specific lanthanide antibodies using C60-SIMS with a 1.1 μm resolution to differentiate cell types. The approach revealed distinct variations in the distribution and intensities of over 150 key ions, including lipids and important metabolites, in different types of tumor microenvironment (TME) cells, such as actively proliferating tumor cells and infiltrating immune cells.^228^ By integrating multiomics profiling, combining lipidomic, metabolomic, and proteomic data, this method provided a more holistic view of the TME. Although it did not achieve segmentation to individual cells, it demonstrated the feasibility of SIMS imaging to integrate multiomics profiling within the context of a complex cellular mosaic, offering valuable insights into the TME’s cellular heterogeneity.

While much of the focus in sc metabolomics for elucidating tumor biology has been on imaging-based mass spectrometry techniques, significant advancements are also being made in nonimaging-based MS methods, broadening the scope of metabolic profiling at the sc level.

A novel approach called sc metabolomics by intact living-cell electro-launching ionization mass spectrometry (sMDA-scM ILCEI-MS) was utilized to study drug action mechanisms in NSCLC cells treated with gefitinib. This method revealed two distinct subpopulations of cells exhibiting differential metabolic responses to the treatment.^124^ A significant number of cells were analyzed across various treatment concentrations, demonstrating the ability of ILCEI-MS to capture subtle metabolic shifts within single cells in response to pharmacological interventions and at relevant throughputs.

Further advancements in ionization technology have led to the development of a novel concentric hybrid ionization source that combines nanoelectrospray ionization with atmospheric pressure chemical ionization (nanoESI-APCI). This technique significantly enhanced the detection of both polar and nonpolar metabolites simultaneously in single cells, improving the limit of detection by an order of magnitude to 10 pg mL^–1^. The method was applied to investigate the metabolic heterogeneity within the human hepatocellular carcinoma tissue microenvironment.^83^ It facilitated the discrimination of cancer cell types and subtypes and explored the metabolic perturbations due to glucose starvation in MCF7 cells as well as the metabolic regulation in cancer stem cells. The study highlighted that the metabolic impacts of glucose starvation were comparable to those observed between different cell types, underlining the profound effects of metabolic conditions on cellular states.

These nonimaging-based MS techniques contribute uniquely to the field of tumor biology by enabling detailed metabolic profiling without the need for spatial mapping, thus providing complementary insights that are crucial for a comprehensive understanding of cellular metabolism. However, such technique will more heavily rely on integrated omics as metabolic heterogeneity needs to be put in the context of cell types and transcriptional states.

### 4.2 Insights from sc Transcriptomics and Proteomics to Understand Tumor Heterogeneity

The sc transcriptomics and sc proteomics have suggested significant metabolic heterogeneity across different cancer types. Future developments can integrate these findings with direct metabolic readouts, which are particularly well-suited for capturing transient cellular states as exhibited under conditions like drug pressure.

Studies focusing on heterogeneity across tumor progression have shown that transcriptional heterogeneity increases with tumor progression and metastasis and is linked to metabolic states. In breast cancer, sc RNA sequencing revealed distinct transcriptional programs associated with metastasis, primarily driven by altered metabolic pathways.^229^ A study on melanoma cells revealed phenotypic heterogeneity and changes in gene expression were closely linked to epigenetic regulation.^230^ In this context, direct metabolic measurements become crucial. Indeed, in breast cancer metabolic heterogeneity in processes like glycolysis, gluconeogenesis, and fatty acid synthesis has been associated with chemotherapy resistance.^231^

When examining heterogeneity between different tumors in different patients, integrated multiomics studies have reported significant cellular heterogeneity. For instance, research on hepatocellular carcinomas (HCC) revealed substantial differences in metabolic and immune activity among patients, emphasizing the necessity for personalized treatment strategies.^232^ Similarly, a study on gastric cancer identified correlations between various metabolic subtypes and levels of immune infiltration, providing valuable insights for customized therapeutic approaches.^233^

In examining spatial heterogeneity, sc transcriptomics of oligodendrogliomas has uncovered significant metabolic differences across tumor regions, highlighting the crucial role of spatial analysis in understanding metabolic diversity.^234^ Furthermore, a prostate cancer study integrating sc transcriptomics with metabolic pathway analysis revealed variations in metabolic activity across different cell types, heavily influenced by mitochondrial function, thus demonstrating extensive spatial heterogeneity and intracell variability.^235^

These and further recent reports^3,221,236,237^ highlight future follow-up validation studies using specialized sc metabolomics analysis, which can elucidate the regulatory mechanisms of individual biochemical pathways and enzymes. This could help identify specific, targetable metabolic vulnerabilities, potentially leading to more effective, tailored anticancer treatments.

## 5 Spaciotemporal Heterogeneities and Cell State Transitions in Development and Diseases

Recent advancements in sc metabolomics provide crucial insights beyond tumor biology, enabling us to understand better the metabolic heterogeneity across spatial and temporal dimensions and in human-relevant diseases or normal physiology (Figure 1).

Development in organisms is governed by the tightly regulated processes of cell proliferation and differentiation, guided by intricate genomic and environmental cues. Sc metabolomics emerges as a pivotal tool for dissecting these complex processes, providing insights into metabolic remodeling and molecular mechanisms during embryonic development across various species. For example, studies on *Xenopus laevis* have highlighted significant metabolic heterogeneity at critical developmental stages, demonstrating the broader applicability and importance of the sc approach in developmental biology.^43,200,238,239^

Leigh syndrome (LS), is a severe neurological disorder characterized by progressive degeneration of the central nervous system, typically presenting in infancy with symptoms such as developmental delay, seizures, and motor skill regression, and is caused by genetic mutations affecting cellular energy production and more specifically severe mitochondrial disease.^240^ It has been suggested that metabolic factors may directly regulate this and related disorders.^241,242^ In LS scRNA sequencing and multiomics analyses in human organoids have demonstrated compromised neuronal morphogenesis in neural progenitor cells (NPCs). These cells remain in a glycolytic proliferative state, preventing proper neuronal differentiation.^243^ Metabolism has been shown to regulate the formation, migration, and differentiation of NPCs.^244−246^ Two major bioenergetic shifts have been identified^247−250^ and disruptions in these transitions were linked to neurological disorders.^241^ Similarly, research using high-throughput sequencing in HCC revealed significant metabolic differences across hematopoietic cell lineages and differentiation stages. Further, glucose transport and metabolism pathways showed significant variation, though the study did not employ sc analysis directly.^251^ The field is now primed for direct insights from sc metabolomics, which will provide precise measurements of altered metabolic states and identify key pathways that could be targeted to modulate disease severity.

Nephrogenesis, the process of kidney formation, involves a significant metabolic transition from glycolysis to fatty acid β-oxidation.^252^ In this regard, MALDI-MSI and scRNA-seq analyses have revealed metabolic cell fate trajectories during kidney differentiation, providing insight into the spatiotemporal dynamics of metabolism.^253^ Researchers extended these findings *in vitro*, enabling them to manipulate metabolic pathways for a better understanding of stem cell differentiation.

In neurodevelopment research, a combination of patch-clamp electrophysiology and capillary electrophoresis-mass spectrometry (CE-MS) enabled the correlation of the physiological activities of neurons with their neurochemical states. This study revealed striking cell-to-cell heterogeneity in neuronal metabolomes and presented an approach applicable to a wider range of smaller cell types.^254^

In brain trauma research, spatial transcriptomics and metabolomics in human brain tissue samples identified molecular markers of metabolic changes, with areas of lipid peroxidation pointing to injured neurons.^255^ This comprehensive approach integrated spatial transcriptomics with MSI for detailed analysis. These efforts could lead to the development of small molecules effective for treating brain trauma, offering advantages over genetic manipulations, which are more challenging to implement in clinical settings.

In further exploring cellular heterogeneities, recent advancements in sc and spatial technologies have provided profound insights into cellular diversity and metabolic states across different tissues. The novel SEAM method combined high-spatial-resolution imaging mass spectrometry with computational algorithms to perform multiscale and multicolor tissue tomography. This approach successfully identified subpopulations of hepatocytes with distinct metabolic features linked to their proximity to fibrotic niches.^191^ The findings were further supported by spatial transcriptomics using Geo-seq, enhancing the understanding of tissue microenvironments at a single-nucleus level. SEAM’s label-free technique, requiring minimal experimental preparation, preserved the samples’ native states and offered substantial throughput. However, the identification of specific cell types relied on subsequent immunofluorescence, complicating the differentiation of cell clusters. Nevertheless, this method revealed that a subset of cells (14.4%) showed unchanged glutathione pathways, a detail obscured in bulk analyses but discernible through sc approaches.

Further extending the exploration of metabolic heterogeneity linked to aging, researchers employed a combination of spatial transcriptomics, sc assay for transposase-accessible chromatin (ATAC)-seq, and RNA-seq alongside lipidomics and functional assays to investigate age-related changes in the male murine liver.^256^ This comprehensive approach uncovered zone-specific and age-related alterations in metabolic states, epigenetics, and transcriptomics. Functionally, it was found that periportal hepatocytes exhibited decreased mitochondrial fitness, while pericentral hepatocytes accumulated significant lipid droplets, indicating distinct metabolic adaptations to aging within the liver. These findings highlight the pivotal role of integrating multiomics techniques to unravel the complex interactions within tissues.

Sc technologies hold further significant promise for aging research.^257^ For example, bioinformatic analyses of sc transcriptomics data have revealed disturbed antioxidant signaling in primate ovarian cells as a hallmark of ovarian aging.^258^ Further studies have indicated upregulation of pro-inflammatory pathways and downregulation of mitochondrial function in the aging lung, suggesting interventions to address aging-related conditions.^259^ In the context of aging, expanding on sc metabolomics studies in addition to other sc techniques could have the added advantage of pointing out specific pathways that are amenable to interventions such es restoring redox state^260^ or supplementing specific nutrients. Such approaches could go beyond current broad recommendations such as calory restriction and exercise.^261^

Though some studies have yet to apply sc metabolomics directly, they serve as a guiding light for future research. Further integrating these sc techniques with sc metabolomics promises to enhance our understanding of temporal and spatial heterogeneities in various diseases, paving the way for innovative treatment approaches.

## 6 Metabolic Heterogeneity Revealed by Whole-Body Non-MS Imaging Techniques

Technologies such as Positron Emission Tomography (PET), Magnetic Resonance Imaging (MRI), and Raman imaging are powerful in reporting cellular or organismal-level metabolic heterogeneities and have significantly enhanced our understanding of various diseases.^262−265^ These and related fluorescence-based technologies vary in their resolution, with some imaging metabolic processes at the sc level while others providing broader spatial insights. Further, these techniques require little sample preparation and can be performed noninvasively and nondestructively. Most importantly, these technique present orthogonal approaches to MS sc metabolomics as they do not suffer from identical technical challenges and limitation. Below, several studies are highlighted as examples of what these technologies can offer. For more comprehensive overview, readers can refer to recent comprehensive reviews.^262−265^

### 6.1 Techniques Providing (Sub)Cellular-Level Metabolic Insights

Non-MS imaging techniques complement metabolic studies.^266^ For example, an alternative approach to directly measuring metabolite concentrations was developed to visualize and quantify multiple enzymatic activities within a native tissue environment. Using a simple fluorescent microscope, this method measured enzymatic activities at saturating substrate conditions, while cell types were identified employing standard immunofluorescent techniques.^267^ The redox-sensitive tetrazolium salt, nitroblue tetrazolium chloride (NBT), served as a detection reagent to monitor product formation of five dehydrogenases over time. This technique enabled the analysis of metabolic interactions between cancer cells and cancer-associated fibroblasts within a breast cancer tissue array. Thus, providing insights into cellular metabolic relationships of single cells in their natural context. A related technique, optical imaging using fluorescent metabolic reporters, monitored metabolic reprogramming in residual breast cancer, revealing increased metabolic flexibility and heterogeneity in persistent disease.^268^ Metabolic states can further be assessed by exploiting the fluorescence lifetimes (FLIM) of intrinsic metabolic cofactors such as NAD(P)H and FAD.^269^ These cofactors exhibit different fluorescence lifetimes depending on their bound (enzyme-associated) or unbound states, reflecting their involvement in metabolic reactions. For example, NAD(P)H and FAD FLIM illuminated metabolic heterogeneity within immune and tumor cells in melanoma, delivering crucial metabolic insights at high resolution.^270^

For monitoring compounds that do not fluoresce, Optical Photothermal Infrared (O-PTIR) imaging can provide direct chemical composition information based on IR absorption. O-PTIR applied to bacterial populations has been employed to analyze phenotypic heterogeneity. By monitoring the Bioplastic poly-3-hydroxybutyrate (PHB) production in *Bacillus* strains, O-PTIR provided a sc biochemical analysis that was crucial for industrial bioprocessing applications where subpopulations of inefficient producer cells can impact productivity.^271^ Similarly, electrochemical methods allow for the monitoring of redox-active metabolites by customizing biosensing interfaces on electrode surfaces to examine a broad spectrum of redox-active compounds. As an example, OptoElecWell-based respiration methodology offered a compact alternative to the widely used Seahorse XF analyzer (sensing respiration rate and glucose consumption), enabling the analysis of yeast metabolism with 20 times fewer samples and was adaptable to single cells. This platform integrated a platinum nanoring electrode for in situ electrochemistry with high-resolution live-cell imaging, allowing simultaneous analysis of basal respiration and intracellular parameters under varying glucose concentrations.^206^

Further advancements in label-free imaging technologies have significantly enriched sc metabolomics by providing high-resolution insights into cellular metabolic states without the use of external markers. Here, Stimulated Raman Scattering (SRS) is a forefront example. Raman spectroscopy is a nondestructive technique to study cellular metabolism with subcellular spatial resolution.

SRS imaging detailed the metabolic profiles of single cells under stress in pancreatic cancer, revealing lipid-rich protrusions that underscore shifts in metabolism during adverse conditions.^1001^ Confocal Raman spectroscopy was employed to quantify biomolecules in NPC cell lines, creating a metabolic map that corroborates findings from UPLC-MS/MS. This technique’s ability to classify cancer versus healthy tissue using machine learning models demonstrates its throughput and application breadth.^272^ Raman combined with deuterium isotope probing (Raman-DIP) and surface-enhanced Raman scattering (SERS) within a microfluidic platform were employed to study bacterial metabolism and multiplexed metabolite analysis at the sc level. Raman-DIP elucidated metabolic adaptations of Salmonella Typhi inside human host cells, highlighting its potential to reveal metabolic changes and heterogeneity among bacterial pathogens.^273^ Meanwhile, the SERS-microfluidic approach used magnetic SERS substrates to analyze metabolites like pyruvate, ATP, and lactate from single cells, revealing metabolic heterogeneity related to cell density and intercell interaction, underscoring the dynamic nature of cellular metabolomics.^274^

Another label-free approach is based on genetically encoded Förster Resonance Energy Transfer (FRET) sensors which allow real-time metabolic analysis at the sc and subcellular level. These sensors typically consist of a bacterial binding protein fused with one or more fluorescent proteins. Upon binding a specific metabolite, the bacterial moiety undergoes a conformational change that alters the fluorescence properties of the sensor. FRET-based sensors, which use two fluorescent proteins, offer the advantage of being ratiometric, making measurements less sensitive to sensor concentration, volume changes, and minor focal drifts compared to single-fluorophore sensors^275^

Several FRET-based sensors have been developed for detecting key metabolites, including ATP, glucose, NADH, glutamate, pyruvate, and lactate. The ATP sensor Perceval, constructed from a circularly permuted GFP and the bacterial regulatory protein GlnK1, allows real-time monitoring of the ATP-ratio in live cells, capturing cellular energy dynamics.^276^ Similarly, a glucose sensor, FLIPglu-170n, uses a bacterial glucose/galactose-binding protein and has demonstrated its ability to measure intracellular glucose concentrations in response to changes in external supply and transporter mutations.^277,278^ NADH sensors such as Peredox have been developed to detect the NADH/NAD (+) ratio, providing insights into cellular redox states, particularly in response to glucose metabolism and electron transport chain activity.^279,280^ These sensors enable quantitative measurements in live cells and subcellular compartments. Similarly, the FLIPE sensor for glutamate measures extracellular glutamate levels, allowing real-time monitoring in live cells, including neurons.^281^ Further, FRET-based sensors have been designed for pyruvate and lactate. A pyruvate sensor was introduced to monitor pyruvate transport, production, and mitochondrial consumption in individual cells, with high temporal resolution, particularly useful for examining neuronal metabolic dynamics.^282^ For lactate, the Laconic sensor distinguished between lactate-producing and lactate-consuming cells, and another lactate/pyruvate sensor, Lapronic, targeted the mitochondrial matrix to assess metabolic fluxes.^283,284^ These sensors provide valuable data on metabolic regulation at the cellular level.

While these technologies offer unique insights into cellular metabolism, they are constrained by the limited number of metabolites they can analyze. For instance, FLIM relies heavily on the fluorescent activities of analytes. FRET-based sensors are also limited by the time required to develop and characterize each sensor, and potential interference from autofluorescence and factors such as pH. While Raman spectroscopy, despite its capability to detect signals from various chemical classes, often struggles with the precise annotation of individual metabolites. These techniques can nonetheless provide a holistic view of cellular function and disease states within a native context. As orthogonal sc analysis methods, they can be multiplexed or performed in parallel to independently confirm the metabolic profiles observed via MS. This complementarity will enhance the reliability of sc data and strengthen the overall conclusions drawn from sc metabolomics studies.

### 6.2 Techniques Providing Insights on Tissue-Level Metabolic Heterogeneity

MRI, computed tomography (CT), and PET are powerful imaging techniques widely used to explore metabolic heterogeneity in various tissues and organ systems frequently applied to studies of metabolic heterogeneity in cancer.^285−287^ These noninvasive methods offer the ability to capture dynamic changes at the cellular and molecular levels within living subjects. MRI utilizes powerful magnetic fields and radio waves to generate detailed images of organs and tissues, excelling in soft tissue contrast. CT employs X-rays to create cross-sectional images, providing excellent spatial resolution for structural details. PET is highly sensitive to metabolic functions, visualizing the distribution of metabolic substrates and products labeled with positron-emitting isotopes. Often used in combination, these techniques do not only complement and validate sc technologies but also motivate further detailed exploration with sc MS technologies.

MRI, particularly multiparametric MRI, demonstrated suitability to detect metabolic changes and provide spatial resolution of metabolic phenotypes. For instance, in clear cell renal cell carcinoma (ccRCC), MRI revealed variability in glycolysis and the TCA cycle, emphasizing significant metabolic differences between and within tumors.^288^ Additionally, hyperpolarized MRI with ^13^C-pyruvate highlighted glycolytic dependencies in hepatocellular carcinoma, providing crucial insights for treatment strategies.^289^ Proton MR spectroscopic imaging (MRSI) is another MRI technique used to detect metabolic differences in tissue components. In glioblastoma, MRSI provided noninvasive metabolic profiling, revealing tumor burden and hypoxia, further highlighting its ability to detect metabolic heterogeneity.^290^

In an example of the capabilities of PET imaging, a detection of the spatial distribution of radioactive tracers within the body, provided a functional overview of metabolic processes in lung adenocarcinoma. PET imaging captured glycolytic heterogeneity, linking it to aggressive histopathological characteristics.^291^ Further, PET imaging combined with nuclear magnetic resonance (NMR) metabolomics in breast cancer revealed metabolic variations between subtypes and across different tumor regions, offering valuable insights into tumor heterogeneity.^292^

Such studies will benefit from further examination using dedicated sc metabolomics technologies. While MRI, CT, and PET imaging offer powerful insights into whole-organism metabolism and underscore the widespread presence of cellular heterogeneities, they are limited by detecting only a few metabolites and operating at lower spatial resolution. In contrast, sc MS and MSI can provide mechanistic understanding by detecting a wider range of metabolites and providing superior resolution. The full scientific potential emerges from employing non-MS imaging techniques in conjunction with sc MS, providing understanding of metabolic processes across various biological scales.

## 7 Future Directions

The recent staggering growth of sc technologies has demonstrated the interest and potential for future applications. To ensure relevant biological heterogeneity is captured robustly, future work should address five key metabolomics-specific goals: (1) quality assurance for the preservation of metabolic states, (2) control and normalization strategies for ion suppression effects, (3) assurance steps for the sample-specific origin of annotated compounds, (4) improvement of multiomics integration, and (5) absolute quantitation of metabolite levels.

To evaluate how sc preparations affect metabolic states it will be critical to establish clear criteria. In this, indicators of energy metabolism or redox state could be especially informative as they can provide dynamic readout for the most likely immediate metabolic perturbations, such as redox stress during cell isolation^99^ or collapse of ATP concentration.^293^ A simple way to validate these could be based on flow cytometry measurements. For example, mitochondrial activity can be read out at a single state level by looking at mitochondrial membrane potential dyes (such as MitoSOX), while ATP levels could be measured with a genetically encoded Förster resonance energy transfer (FRET)-sensor.^294^ Beyond these, orthogonal approaches, as discussed in section 6, can validate certain measurements as for example NAD perturbations. Further controls could compare bulk to pooled sc samples. Pooled sc samples would be prepared by first isolating single cells and then pooling them together. These can then be analyzed in parallel to bulk isolated cells to highlight significant perturbations. Here too controlling for indicators of energy and redox states, such as ATP/ADP ratios, NAD/NADH ratios, or the oxidized to reduced glutathione ratio will be informative. Of note, because of the dynamic nature of these perturbations, it is unlikely that these can be captured at sc transcriptomics or proteomics levels. Overall, the rigor demanded of metabolomics sc isolation is, and should be, higher than for other sc omics technologies.

Similar considerations could address ion suppression effects in future applications, emphasizing the pursuit of quantitative over qualitative readouts for accurate interpretation of cellular metabolic states. Such quantitative metabolic readouts will enable better orthogonal integration and control—through links to genetic or proteomic effects or through independent metabolic measurements—and allow for more robust benchmarking and validation. For instance, using independent biological replicates rather than just increasing the number of individual cell measurements can more effectively distinguish true biological variability from technical noise.^88^ In addition, the effective use of labeled internal standards and isotope ratio analysis could be used to validate at the very least individual key metabolic changes.^77,95^ Extending this to whole labeled metabolomes offers a potential alternative approach.^295,296^ Altogether these steps will ensure that observed differences genuinely reflect metabolic states rather than stem from measurement artifacts or inconsistencies.

To truly benefit biological research, sc metabolomics techniques must now move from solving isolated technical challenges to developing approaches with a holistic view that considers their integration into broader biological goals and contexts. Indeed, we can distinguish cell types using a simple light microscope and sc metabolomics technologies should aim for comparisons beyond pseudobulk analysis. The sc metabolomics conditions and controls used in method development should be aimed at finding key transient metabolites reporting heterogeneity among otherwise closely related perhaps transcriptionally similar cell populations. In this respect metabolic perturbations (by drugs or genetic rescues with enzyme-dead mutants) could be more informative. For example, cells treated with a drug can be mixed post treatment in different ratios and then the expected quantitative changes observed using the sc methodology. This will ensure that novel sc metabolomics technologies can provide a layer of information not accessible through sc transcriptomics or sc proteomics approaches.

The establishment of community standards in proteomics, including standardized protocols, data formats, quality control measures, and minimum information requirements for reporting experiments, has significantly improved reproducibility and comparability across studies.^297^ Recommendations have been proposed specifically for sc proteomics as well, though they are still awaiting broad community adoption.^298^ Likewise, we should aim to implement such standards in sc metabolomics to enhance consistency, reliability, and reproducibility across experiments and laboratories. These recommendations and standardizations will help ensure the broader adoption of novel sc metabolomics methodologies within the research community.

## 8 Conclusion

In the evolving field of sc metabolomics, technological advancements continue to enhance our ability to probe cellular heterogeneity with improving scope and precision. However, challenges such as ionization effects, normalization issues, and the assurance that the measured molecules genuinely reflect biological sources and states remain significant hurdles. We need to push for quantitative readouts, validation of metabolic differences, and preservation of metabolic states. Finally, relevant biological questions should drive the technological developments, ensuring we move beyond simple comparisons between different cell types and demonstrate genuine ability to access information beyond bulk measurements.

## Abbreviations

AFADESI-MSI: ambient fiber-assisted desorption electrospray ionization mass spectrometry imaging
Aβ: amyloid-beta
APCI: atmospheric pressure chemical ionization
ATAC: assay for transposase-accessible chromatin
CAMI: computer-assisted microscopy isolation
ccRCC: cell renal cell carcinoma
CE: capillary electrophoresis
CE-MS: capillary electrophoresis-mass spectrometry
CT: computer tomography
DBDI: dielectric barrier discharge ionization
DBTL: design-build-test-learn
DHB: 2,5-dihydroxybenzoic acid
DIA: data-independent acquisition
DIA-PASEF: data independent acquisition parallel accumulation-serial fragmentation
DIMS: direct infusion mass spectrometry
FACS: fluorescence-activated cell sorting
FASI: field amplified sample injection
FI-MS: flow injection mass spectrometry
f-LAESI: fiber-based laser ablation electrospray ionization
FLIM: fluorescence lifetimes
FRET: Förster resonance energy transfer
FTICR: Fourier transform ion cyclotron resonance
FT-ICR MSI: Fourier transform ion cyclotron resonance mass spectrometry imaging
GC-MS: gas chromatography-mass spectrometry
HCC: hepatocellular carcinomas
HeLa: Henrietta Lacks
HER2: human epidermal growth factor receptor 2
HRMS: high-resolution mass spectrometry
ILCEI-MS: intact living-cell electrolaunching ionization mass spectrometry
IM: ion mobility
IR-MALDESI-MS: infrared matrix-assisted laser desorption electrospray ionization mass spectrometry
LAAPPIMS: infrared laser ablation atmospheric pressure photoionization mass spectrometry
LACFI-MSI: laser-assisted carbon fiber ionization
LAESI-MS: laser-ablation electrospray ionization mass spectrometry
LAESI-IMS-MS: laser-ablation electrospray ionization ion-mobility mass spectrometry
LCM: with laser capture microdissection
LC-MS: liquid chromatography-mass spectrometry
LDIS: large-volume dual preconcentration by isotachophoresis and stacking
LEM: laser etching guided droplet microarray
LS: Leigh syndrome
MALDI-MS: matrix-assisted laser desorption/ionization mass spectrometry
MCF7: Michigan Cancer Foundation-7
MFD-MD: multicolor fluorescence detection-based microfluidic device
ML: machine learning
MM: multiple myeloma
MMS: multimodal Mass Spectrometry
MRI: magnetic resonance imaging
MRSI: proton MR spectroscopic imaging
MS: mass spectrometry
MSI: mass spectrometry imaging
nanoCEMS: nano capillary electrophoresis-mass spectrometry
nanoESI: nano electrospray ionization
NBT: nitroblue tetrazolium chloride
NIMS: nanostructure imaging mass spectrometry
NMR: nuclear magnetic resonance
NPC: neural progenitor cells
NSCLC: non-small cell lung cancer
O-PTIR: optical photothermal infrared
PANC-1: Pancreatic Carcinoma-1
PET: positron emission tomography
PHB: poly-3-hydroxybutyrate
PTR: proton-transfer-reaction
Raman-DIP: deuterium isotope probing Raman
Sc: single-cell
SCM: spatial coherence measure
SCP-LVC-MS: sc printer technology combined with liquid vortex capture-MS
scSpaMet: single cell spatially resolved metabolic
SERS: surface-enhanced Raman scattering
SFE: supercritical fluid extraction
SIMS: secondary ion mass spectrometry
sMDA-scM ILCEI-MS: sc metabolomics by intact living-cell electro-launching ionization mass spectrometry
SRS: stimulated Raman scattering
STC-DESI: segmented temperature-controlled desorption electrospray ionization
SWATH: sequential window acquisition of all theoretical mass spectra
TOF: time-of-flight
t-AP-LDI: transmission ambient pressure laser desorption ionization
3D-SMF: 3D spatially resolved metabolomic profiling framework
UEg-MSI: ultrasound elastography with mass spectrometry imaging
UPLC-MS: ultrahigh performance mass spectrometry
